# When Your Doctor “Gets It” and “Gets You”: The Critical Role of Competence and Warmth in the Patient–Provider Interaction

**DOI:** 10.3389/fpsyt.2019.00475

**Published:** 2019-07-04

**Authors:** Lauren C. Howe, Kari A. Leibowitz, Alia J. Crum

**Affiliations:** ^1^Department of Business Administration, University of Zurich, Zurich, Switzerland; ^2^Department of Psychology, Stanford University, Stanford, CA, United States

**Keywords:** placebo effects, placebo response, patient–provider interactions, warmth, competence, provider characteristics, provider demeanor

## Abstract

**Background:** Research demonstrates that the placebo effect can influence the effectiveness of medical treatments and accounts for a significant proportion of healing in many conditions. However, providers may differ in the degree to which they consciously or unconsciously leverage the forces that produce placebo effects in clinical practice. Some studies suggest that the manner in which providers interact with patients shapes the magnitude of placebo effects, but this research has yet to distill the *specific* dimensions of patient–provider interactions that are most likely to influence placebo response and the mechanisms through which aspects of patient–provider interactions impact placebo response.

**Methods:** We offer a simplifying and unifying framework in which interactions that boost placebo response can be dissected into two key dimensions: patients’ perceptions of *competence*, or whether a doctor “gets it” (i.e., displays of efficiency, knowledge, and skill), and patients’ perceptions of *warmth*, or whether a doctor “gets me” (i.e., displays of personal engagement, connection, and care for the patient).

**Results:** First, we discuss how this framework builds on past research in psychology on social perception of competence and warmth and in medical literature on models of effective medical care, patient satisfaction, and patient–provider interactions. Then we consider possible mechanisms through which competence and warmth may affect the placebo response in healthcare. Finally, we share original data from patients and providers highlighting how this framework applies to healthcare. Both patient and provider data illustrate actionable ways providers can demonstrate competence and warmth to patients.

**Discussion:** We conclude with recommendations for how researchers and practitioners alike can more systematically consider the role of provider competence and warmth in patient–provider interactions to deepen our understanding of placebo effects and, ultimately, enable providers to boost placebo effects alongside active medications (i.e., with known medical ingredients) and treatment in clinical care.

## Introduction

The doctor has been called “a powerful therapeutic agent” (p. 1,067) (1) who can evoke healing in her or his patients even by simply interacting with them. One way providers can help their patients heal, and the focus of this paper, is through eliciting *placebo effects*, or “healing that is produced, activated, or enhanced by the context of the clinical encounter, as distinct from the specific efficacy of treatment interventions” (2). Diverse factors can produce placebo effects, including medical rituals (e.g., taking a pill) and provider behaviors (e.g., communication). For example, providers explicitly stating to patients that a treatment will improve their condition makes it more likely that the treatment will do so (3, 4). Placebo effects bolster the efficacy of both active medications (5–7) and treatments with no active medical properties, ranging from sugar pills (8) to inert creams described as pain relievers (9) to sham acupuncture involving fake needles that never pierce the skin (10).

But not all placebo effects are created equal. A series of studies suggests that how providers interact with their patients shapes the magnitude of placebo effects (10–13). But while these studies acknowledge that patient–provider interactions are critical to placebo response, they do not provide a theoretical framework for the *specific* dimensions of the patient–provider interaction that enhance placebo effects and thus shape a patient’s physical health outcomes.

In the current article, we address four key questions, which correspond to the four main sections of the article:

1. What are the key dimensions of patient–provider interactions?
2. In what ways do these dimensions moderate placebo response?
3. What are the mechanisms through which these dimensions moderate placebo response?
4. How can providers leverage these dimensions deliberately in clinical care?

In considering these questions, we delineate a novel framework proposing that interactions that boost placebo response can be dissected into two key dimensions: patients’ perceptions of *competence*, or whether a doctor “gets it” (i.e., displays of efficiency, knowledge, and skill) and patients’ perceptions of *warmth*, or whether a doctor “gets me” (i.e., displays of personal engagement, connection, and care for the patient). We suggest that competence and warmth work together to influence placebo response and therefore shape effective healthcare.

## What Are the Key Dimensions of Patient–Provider Interactions?

Is there a parsimonious way to represent the many diverse qualities that may be present in patient–provider interactions? We tackle this question in three steps. First, we discuss the psychological literature on social perception, which identifies key dimensions that underlie our impressions of others. Second, we introduce a model of patient–provider interactions that explains how key dimensions from social perception apply in the healthcare context. Third, we illustrate how these key dimensions are evident in the medical literature on patient–provider interactions by reviewing theoretical and empirical work on effective patient–provider interactions.

### Competence and Warmth: Two Core Dimensions of Social Perception

Psychologists have long been interested in understanding the dimensions on which people judge others when forming first impressions. In order to successfully navigate one’s social world, a person must constantly and rapidly make accurate assessments of other people. Should a stranger be approached or avoided? Is a person a suitable friend or romantic partner? Is an expert worthy of trust? To answer such questions, people need to quickly determine whether another person is likely and able to harm or help them. Although many dimensions for the factors that underlie such social judgments have been proposed, over 50 years of research suggests that they can all be distilled into two key dimensions: warmth and competence (14–20).

One study attempting to identify the underlying dimensions of personality asked participants to describe different people they knew by selecting personality traits from a list of over 60 different traits (21). These researchers then evaluated the degree to which these traits co-occurred in people’s descriptions of a particular person. They found the traits that co-occurred frequently could be grouped into those that described intellectual qualities that were either good or bad (i.e., competence—e.g., qualities like determined and industrious *vs*. irresponsible and unintelligent) and social qualities that were good or bad (i.e., warmth—e.g., qualities like sincere and good-natured *vs*. irritable and humorless). These two dimensions were independent and accounted for most of the variance in people’s judgments of others.

In other research, participants generated descriptions of events that helped them form strong impressions of other people or themselves (22). Of the over 1,000 descriptions generated by these participants, approximately three-fourths depicted considerations of warmth or competence, as rated by independent judges. In yet another study, a pool of 200 diverse traits were rated on a variety of dimensions, including the degree to which they captured warmth and captured competence (23). These ratings of a trait’s warmth and competence predicted all but 3% of the variance in ratings of trait favorability, suggesting that these two ingredients are key to describing positive and negative qualities in person perception.

Together these studies, and dozens of others using a variety of methodologies, suggest that warmth and competence are two key dimensions holding the greatest explanatory power when it comes to positive and negative evaluations of others.^1^ Qualities like friendliness, honesty, trustworthiness, good-naturedness, empathy, and kindness (*vs*. coldness, deceit, and unreliability) are all essentially different ways to describe a person’s general warmth. Qualities like intelligence, power, assertiveness, ambition, efficacy, and skill (*vs*. inefficiency, indecisiveness, passivity, and laziness) are all essentially different ways to describe a person’s general competence (15, 20). Though these dimensions have sometimes been called by other names [e.g., agency and communion (25–27); for a review see Ref. (17)] regardless of the nomenclature, there is remarkable consistency among researchers in the qualities that are commonly reflected by these two dimensions.

There is a strong evolutionary argument for the primacy of warmth and competence: the need to rapidly determine whether a person intends to, and is capable of, harming or helping an individual. Essentially, warmth encapsulates answers to the question of “Are this person’s intentions toward me positive or negative?” and competence encapsulates answers to the question of “Does this person have the ability to enact those positive or negative intentions?” (14). To promote survival, a person must be able to find an answer to these key questions whenever they encounter someone new.

And indeed, people make these judgments rapidly and non-consciously, any time they evaluate someone new. People judge others as warm or competent based on even brief exposure to another person’s behavior (28–30). For example, both adults and children form evaluations of warmth and competence after brief, 100-millisecond exposure to a person’s face (31, 32). These two dimensions are readily perceived from a variety of limited non-verbal information, such as tone of voice, body posture, and facial expressions (33–35). Further, ratings of warmth predict liking and ratings of competence predict respect for others (25, 36). Warmth and competence thus seem likely to influence both the quality and outcomes of a variety of important interpersonal interactions, including patient–provider interactions.

In summary, decades of research in social, evolutionary, and cognitive psychology have shown that a multitude of qualities can essentially be distilled into the two core dimensions of competence and warmth, and that these dimensions are fundamental to how people form impressions of others. Next, we apply this competence and warmth framework to healthcare.

### Judgments of Competence and Warmth in Healthcare: The Provider “Gets It” and “Gets Me” Framework

Patients’ assessments of a provider likely also follow these two key dimensions of social perception, but with a slightly different flavor. We propose a healthcare-specific framework in which patients assess competence by judging whether the provider “gets it” (i.e., demonstrates efficiency, knowledge, and skill) and assess warmth by judging whether the provider “gets me” (i.e., demonstrates personal engagement, connection, and care for the patient; in other words, whether a provider sees a patient as a social being, and not just in terms of their health or illness). See **Table 1** for a summary of these dimensions.

**Table 1:** Judgments of competence and warmth in healthcare: the provider “gets it” and “gets me” framework.

	Competence: “My provider gets it”	Warmth: “My provider gets me”
**Definition**	Patient perceptions of *competence*, i.e., displays of efficiency, knowledge, and skill	Patient perceptions of *warmth*, i.e., displays of personal engagement, connection, and care for the patient
**Key question in assessments of this dimension**	Does the provider understand the diagnosis, treatment, and procedures?	Does the provider understand me as a person?
**Examples of general qualities**	Education, diagnostic ability, general medical and procedural knowledge, confidence, articulateness, clarity of explanations, use of technology	General friendliness and social engagement (e.g., smiling, making eye contact), introducing themselves, being polite to co-workers
**Examples of patient-specific qualities**	Knowledge of patients’ family history, experience with similar patients, answering patients’ specific questions and concerns	Knowledge of the patient as a person (i.e., outside of the healthcare context), understanding of patient values, active listening, feeling that the provider respects and does not judge the patient
**Qualities bridging warmth and competence**	Use of patient-friendly language, individualization of patient explanations and/or care, engagement of patients in their own care and/or decision-making	

We define patient-specific qualities as providers’ qualities, such as knowledge of important aspects of a patient’s life outside of the healthcare context (warmth) and experience working with similar patients (competence), that reflect knowledge of the specific patient’s individual needs, desires, and/or perspectives, as opposed to more general qualities of providers, such as general friendliness (warmth) and general medical knowledge (competence), that do not necessarily require knowledge of the specific patient’s individual needs, desires, and/or perspectives.

When assessing whether a provider “gets it,” a patient may pay attention to cues indicating whether a provider has the necessary qualities to conduct relevant procedures, make an accurate diagnosis, and make the best recommendations for treatment. When assessing whether a provider “gets me,” a patient may pay attention to cues indicating whether a provider recognizes and respects that this individual is a person with a life outside of the healthcare context who has their own desires, needs, and values.

There are a multitude of qualities that could bolster patients’ perceptions that a provider “gets it,” all of which involve a practitioner’s perceived expertise and ability to help address a patient’s medical concerns. Some qualities might foster perceptions of medical competence in a broader sense, such as whether a provider attended a top-tier medical school, if they seem up-to-date on medical research, or if they speak clearly and confidently. Other qualities might instead focus on perceived competence regarding the patient and their particular situation. For example, does a patient feel like the provider knows their family history, has experience with patients who are similar to them, and can answer their specific questions?

Similarly, patients’ perceptions that a provider “gets me” could be cultivated in different ways. Some ways involve very general qualities or actions: whether the provider smiles at and sits near the patient, whether they introduce themselves and use the patient’s name, and even whether they are polite to their co-workers at the hospital. These qualities and behaviors, as signals of general positive social engagement, may foster the perception that a provider is likely to regard their patient as a social being worthy of human dignity and respect. Cultivating perceived warmth could also involve qualities that are more patient-specific: listening to a patient and acknowledging their individual perspectives, asking a patient questions about their life outside of the healthcare context to get to know them as a person, appearing to understand the social world of the patient and their values, and respecting the patient. Warmth may also encompass interpersonal skills that bolster perceptions of a provider’s engagement with and care for the patient (e.g., active listening) as well as their emotional feelings toward the patient (e.g., empathy).^2^

### Competence and Warmth in the Medical Literature

We have proposed that patient–provider interactions can be distilled into two key dimensions: whether a provider appears to “get it” (i.e., competence) and “get me” (i.e., warmth). Here we describe how these dimensions, although not always explicitly categorized as such, represent the foundation of existing theories of effective medical care.

#### Competence and Warmth in Theoretical Models of Medical Care

Competence and warmth surface as two key dimensions in a variety of theoretical models of effective medical care, as outlined in **Table 2**. Major advances in our understanding of medicine have often involved a shift from considering only a provider’s competence as critical to patient care to also incorporating a provider’s warmth.

**Table 2:** Competence (provider “gets it”) and warmth (provider “gets me”) in theories of medical care.

Competence/“gets it”	Warmth/“gets me”	References
**Biomedical:** need to know the illness	**Biopsychosocial:** need to know the person who has the disease	Engel (40); McCormick (41); Smith and Hoppe (42)
**Physician-centered medicine:** focuses on the doctor’s interpretation of the evidence and diminishes the importance of human relationships and the role of the patient	**Patient-centered medicine:** focuses on recognizing patients’ individual perspectives and taking them into account in medical care	Bensing (43); Brown et al. (44); Grol et al. (45); King and Hoppe (46); Levenstein et al. (47); Smith and Hoppe (42); Sweeney et al. (48)
**Voice of medicine:** technical aspects, symptoms, and the etiology and treatment of specific diseases	**Voice of the lifeworld:** viewing problems in patients’ personal and sociocultural context	Mishler (49)
**A question of facts:** whether physicians possess the technical expertise necessary for care	**A question of personal values:** whether a treatment resonates with patients’ preferences (e.g., lifestyle, health beliefs, goals)	Eddy (50)
**Need to know and understand:** a provider’s scientific role	**Need to feel known and understood:** a provider’s caring role	Engel (51)
**Cure-oriented:** problem-solving (e.g., asking the patient questions and providing them with information)	**Care-oriented:** reducing patient anxiety (e.g., by using empathy, paraphrasing)	De Valck et al. (52); Van Dulmen and Van Den Brink-Muinen (53)
**Instrumental/task-oriented:** target diagnosis and treatment	**Affective/socio-emotional oriented:** target rapport and relationship building	Bensing et al. (54); Roter and Larson (55)
**Scientific ability/competence**	**Patient narratives:** listen to patient stories, engage empathetically, take patients’ perspectives	Charon (56)

One of the earliest calls to incorporate warmth into models of medical care was the shift from biomedical to biopsychosocial models of medicine (40–42). Biomedical models focused on tasks related to medical competence: rooting out physical causes of illness, using diagnostic tests to determine treatment, and intervening at the level of biology. Biopsychosocial models emphasized the critical role of psychological factors (e.g., personality, mood, coping skills) and social context (e.g., culture, family, socioeconomic status) in health. Biopsychosocial models thus encouraged a greater focus on patients’ concerns, comfort, values, and goals—the “getting me” of medicine.

The role of warmth alongside competence is further reflected in the shift from a doctor-centered, physician-centered, or disease-centered approach (43, 45, 48) to patient-centered medicine (44, 46, 47). As Levenstein and colleagues (47) suggested, in patient-centered medicine “the task of the physician is twofold, to understand the patient and to understand the disease” (p. 24). Patient-centered medicine suggests that most effective treatments based on exceptional knowledge (the “getting it” of medicine) may prove irrelevant if these treatments do not align with a patient’s values and desires, which requires recognizing the patient as a social being and putting effort into “getting me.” Similarly, other research distinguishes between disease as objective (i.e., abnormalities of the structure and function of body organs and systems) and illness as subjective, e.g., incorporating how a patient perceives the event and how it affects their life (57).

There are similar parallels in the “voice of the lifeworld” and the “voice of medicine” (49), or as “a question of facts” *versus* “a question of personal values” (50), as described in **Table 2**. Engel captured these dimensions neatly as two different patient considerations: the *need to know and understand* and the *need to feel known and understood* (51)*.* A quote from Engel encapsulates the importance of a provider’s warmth as well as competence:

For the patient, to feel understood by the physician means more than just feeling that the physician understands intellectually, that is, ‘comprehends’ what the patient is reporting and what may be wrong, critical as these are for the physician’s scientific task. Every bit as important is that the physician display understanding about the patient as a person, as a fellow human being, and about what he is experiencing and what the circumstances of his life are. (p. 11)

Later models captured competence and warmth as behaviors that are *cure-oriented versus care-oriented* (52, 53), *instrumental versus affective* (54), and *task-oriented versus socio-emotional* behaviors (55). The tradition of narrative medicine (56) suggested directly that “a scientifically competent medicine alone” (p. 1,897) is not sufficient for effective healthcare. This tradition argues that physicians must complement their scientific ability by listening to patients’ stories, engaging with them empathetically, and understanding their individual perspectives. By acknowledging the role of personal connections between providers and patients in healthcare, this tradition, as well as the substantial interest in empathy (58, 59) and the emotional aspects of patient–provider communication (60) in the medical literature in recent years, moved medicine closer still toward recognizing the importance of warmth.

In the medical literature, the past decades have involved a shift from a focus on “getting it” to a focus on also “getting the patient.” However, often in these models, warmth and competence have been portrayed as in conflict or competition, or as alternative rather than complementary approaches to care. We propose, and the social perception literature supports, that there need not be a trade-off between warmth and competence, and that these two dimensions often bolster one another.

#### Competence and Warmth in Medical Research on and Measures of Patient Satisfaction

Next, we review some of the most highly-cited measures of patient satisfaction to illustrate that the competence and warmth framework can distill the provider characteristics present in these measures. As can be seen in **Table 3**, widely-used patient satisfaction scales such as the Press Ganey Survey (61) and the Hospital Consumer Assessment of Healthcare Providers and Systems (HCAHPS) (62) capture both warmth (e.g., is courteous) and competence (e.g., is prompt). While these patient satisfaction scales may have their flaws, they nevertheless implicitly assess both competence and warmth, demonstrating that these dimensions are already considered important to effective healthcare.

**Table 3:** Competence and warmth in items from patient satisfaction scales commonly utilized in clinical care evaluations (the Press Ganey Survey and Hospital Consumer Assessment of Healthcare Providers and Systems).

Press Ganey Outpatient Medical Practice Survey: seven relevant items, out of 10 items	

**Items associated with competence**	**Items associated with warmth**
Explanations the care provider gave you about your problem or condition	Friendliness/courtesy of the care provider
Information the care provider gave you about medications (if any) Instructions the care provider gave you about follow-up care (if any)	Concern the care provider showed for your questions or worries
**Items bridging competence and warmth**	
Degree to which care provider talked with you using words you could understand	
Care provider’s efforts to include you in decisions about your treatment	
**Hospital Consumer Assessment of Healthcare Providers and Systems (HCAHPS): seven out of seven items**	
**Items associated with competence**	**Items associated with warmth**
After you pressed the call button, how often did you get help as soon as you wanted it?	How often did (nurses/doctors) treat you with courtesy and respect?
	How often did (nurses/doctors) listen carefully to you?
**Items bridging competence and warmth**	
How often did (nurses/doctors) explain things in a way you could understand?	

Competence and warmth also underlie the constructs captured in some of the most highly cited scales used in medical research (from citations from Google Scholar in November 2018), including the Risser Patient Satisfaction Scale (63) (>490 citations), the Picker Patient Experience Questionnaire (64) (PPE-15, >440 citations), the Medical Interview Satisfaction Scale (65) (MISS, >440 citations, the Consultation Satisfaction Questionnaire (66) (CSQ, >410 citations), and the La Monica-Oberst Patient Satisfaction Scale (67) (LOPSS, > 280 citations), as well as more recently devised scales of patient satisfaction (e.g., the Short Assessment of Patient Satisfaction (SAPS) scale) (60) (see **Table 4** and **Supplemental Table 1**). For example, the items in the LOPSS (67) capture warmth (e.g., is pleasant and gentle) and competence (e.g., is thorough and efficient).

**Table 4:** Competence and warmth in items from patient satisfaction scales developed for medical research.

La Monica-Oberst Patient Satisfaction Scale (LOPSS): sample of 24 relevant items, out of 41 items	

**Items associated with competence**	**Items associated with warmth**
Should be more thorough (R)	Is not as friendly as (s)/he should be (R)
Seems disorganized and flustered (R)	Makes me feel like a “case,” not an individual (R)
Does not follow through quickly enough (R)	Seems more interested in completing tasks than listening to concerns (R)
Tells me what treatment effects to expect	I can share my feelings when I need to talk.
Seems to know what s/he is talking about	Does things to make me feel more comfortable
Would know what to do in an emergency	Is gentle in caring for me
Appears to be skillful at her/his work	Treats me with respect
Makes helpful suggestions	Appears to enjoy caring for me
Gives complete explanations	Is pleasant to have around
**Items bridging competence and warmth**	
Neglects to be sure I understand importance of my treatments (R)	
Acts like I cannot understand the medical explanation of my illness (R)	
Fails to consider my opinions and preferences regarding plans for my care (R)	
Helps me to understand my illness	
Gives directions at just the right speed	
Shows me how to follow my treatment program	
**Risser Patient Satisfaction Scale: sample of 15 relevant items, out of 25 items**	
**Items associated with competence**	**Items associated with warmth**
*Technical-professional area (seven items)*	*Trusting relationship area (11 items)*
The nurse really knows what s/he is talking about.	The nurse is understanding in listening to a patient’s problems.
The nurse is not precise in doing his/her work. (R)	The nurse should be more friendly than s/he is (R).
The nurse is too slow to do things for me. (R)	I’m tired of the nurse talking down to me. (R)
The nurse is skillful in assisting the doctor with procedures.	The nurse is a person who can understand how I feel.
The nurse is often too disorganized to appear calm. (R)	The nurse is pleasant to be around.
**Items bridging competence and warmth**	
*Educational relationship area (seven items)*	
The nurse gives directions at just the right speed.	
I wish the nurse would tell me about the results of my tests more than s/he does. (R)	
It is always easy to understand what the nurse is talking about.	
The nurse explains things in simple language.	
Too often the nurse thinks you can’t understand the medical explanation of your illness, so s/he just doesn’t bother to explain. (R)	

(R) indicates that the item describes a provider who is lower on warmth or lower on competence. Otherwise, the item is representative of higher warmth or higher competence. Some other items in these scales not captured in this table assessed general satisfaction and/or confidence in providers, which may be shaped by perceptions of both warmth and competence.

Many critical capabilities of providers highlighted in these measures of patient satisfaction rely on both competence and warmth. For example, the Press Ganey Survey assesses the degree to which a provider made efforts to include the patient in decisions about treatment. To effectively engage a patient in the treatment process, a provider needs the competence to advise a patient on the technical aspects of care and to know what treatment options are suitable. But a provider also needs warmth to gain insight into a patient’s perspective and values in order to present relevant options to a patient. They need warmth to judge a patient’s knowledge and skills appropriately based on their life experiences and to take that into account when conveying information to them. And, they need warmth to cultivate enough approachability to make a patient feel comfortable engaging in their care. Abilities such as advice-giving may function similarly. Of course, a provider needs the competence to know possible recommendations and to explain them clearly to patients, but a provider also needs the warmth to choose advice that is appropriate for a particular patient and to relate it to the patient to encourage adherence. Competence and warmth combined thus form the foundation of many healthcare skills, as highlighted in **Tables 3** and **4**.

Several scales (i.e., Press Ganey, CSQ, MISS, SAPS) include questions assessing how satisfied patients were with the amount of time that their provider spent with them. Some research shows that provider warmth shapes perceptions of the time spent with a provider during a medical exam (68), and so measures of patient satisfaction with visit length may be linked with perceived provider warmth.

Thus, when attempting to measure the quality of interactions with providers, existing scales tap into the core dimensions of competence and warmth or assess skills that require both of these dimensions. Details on the validity of these scales are reported elsewhere (69–71). Here we focus primarily on the fact that all of these scales capture the core dimensions of competence and warmth, therefore providing further evidence that a combination of these qualities are critical to effective healthcare (in this case, as evidenced by patient satisfaction).

#### Competence and Warmth in Medical Research on and Measures of Patient–Provider Interactions

Research-based measurements of patient–provider interactions also illuminate the core dimensions of competence and warmth (see **Table 5**). Some widely used methods for analyzing patient–provider interactions include the Roter Interaction Analysis System (55, 72) (RIAS, >700 citations), a coding systems for patient–provider communication, and the coding scheme associated with the Four Habits model (73, 74) (>190 citations).

**Table 5:** Warmth and competence in behaviors from the Roter Interaction Analysis System and Four Habits Coding Scheme used to code dialogue between patients and providers.

Roter Interaction Analysis System	
**Behaviors associated with competence**	**Behaviors associated with warmth**
Providing biomedical information (e.g., about medical condition or therapeutic regimen)	Positive talk (e.g., jokes and laughter, approval, compliments)
Orientation (e.g., providing directions and instructions)	Negative talk (e.g., disagreements, disapproval and criticisms) (R)
Providing information about lifestyle and self-care	Social talk (i.e., non-medical chit-chat) Emotional talk (e.g., reassurance, empathy)
Asking questions about medical condition or therapeutic regiment	Asking questions about psychosocial topics
Behaviors bridging competence and warmth	
Partnering and activation (e.g., asking for patient opinions, asking for patient understanding)	
Four Habits Coding Scheme	
**Behaviors associated with competence**	**Behaviors associated with warmth**
Clinician indicates clear familiarity with patient’s history/chart (e.g., mentions recent tests performed).	Patient is greeted in a manner that is personal and warm (e.g., clinician asks patient how s/he likes to be addressed, uses patient’s name).
The clinician attempts to elicit the full range of the patient’s concerns by generating an agenda early in the visit.	Clinician makes non-medical comments, using these to put the patient at ease.
Clinician fully/clearly explains the rationale behind current, past, or future tests and treatments.	Clinician openly encourages/is receptive to the expression of emotion (e.g., through use of continuers or appropriate pauses).
Clinician fully explores barriers to implementation of treatment plan.	Clinician displays non-verbal behaviors that express great interest, concern, and connection (e.g., eye contact, tone of voice, and body orientation) throughout the visit.
**Behaviors bridging competence and warmth**	
Clinician shows great interest in exploring the patient’s understanding of the problem (e.g., asks the patient what the symptoms mean to him/her).	
Clinician attempts to determine in detail/shows great interest in how the problem is affecting the patient’s lifestyle (work, family, daily activities).	
Clinician clearly encourages and invites paint’s input into the decision-making process.	

(R) indicates that the measure describes a provider who is lower on warmth or lower on competence. Otherwise, the measure is representative of higher warmth or higher competence.

The RIAS categorizes dialogue into two buckets: 1) task-focused behaviors, involving gathering data to determine care and providing patient education and counseling, and 2) affective behaviors, involving building a relationship and rapport with patients and responding to a patient’s emotions. Task-focused behaviors often reflect competence, such as asking questions about a medical condition, discussing the results of tests, and giving instructions about treatment. Affective behaviors reflect warmth, such as emotional expressions toward the patient (e.g., concern, optimism, reassurance), verbal attentiveness (e.g., paraphrasing, empathy), social behaviors (e.g., making personal remarks, joking, laughter), and negative talk (e.g., expressing disapproval or criticism) (75–77). The Four Habits model focuses on developing four key families of skills in providers, namely investing in the beginning of the visit, eliciting patient perspectives, demonstrating empathy, and investing in the end of the visit (73, 74). Many of the skills in the model involve warmth (e.g., create rapport quickly, make at least one empathic statement) and many involve competence (e.g., deliver diagnostic information, provide education). As with the patient satisfaction scales, some measures in these scales build on both competence and warmth (e.g., dispensing advice relevant to a patient’s lifestyle, checking patients’ understanding, and encouraging patients to talk).

Provider empathy has raised much recent interest, particularly given its association with improved patient health outcomes (78–81). One of the most widely used scales of provider empathy is the 20-item Jefferson Scale of Physician Empathy (80) (>600 citations), which essentially assesses to what degree providers personally endorse the importance of “getting the patient”; for example, items include whether a provider agrees that “Physicians’ understanding of their patients’ feelings and the feelings of their patients’ families is a positive treatment factor” and “It is as important to ask patients about what is happening in their lives as it is to ask about their physical complaints.” To some degree, these items assess providers’ beliefs about whether warmth is relevant to a provider’s competence (e.g., whether it is an important part of diagnosis and treatment). These qualities seem likely to bolster perceptions of a provider’s warmth.

Echoing measures of patient satisfaction, other research-based measures that dissect patient–provider interactions (e.g., dialogue) into important qualities again capture the core dimensions of competence and warmth.

#### Competence and Warmth in Experimental Research on Patient–Provider Interactions

Some studies have experimentally compared more standard interactions (e.g., meeting basic standards for clinical care, but limiting the social aspects of the interaction) with “enhanced” interactions that focus more on building rapport and positive engagement with a patient. The qualities in these studies can also be organized into the competence and warmth framework. Some manipulations involve verbal statements that indicate competence or warmth explicitly, and others tap into non-verbal behaviors that signal competence and warmth.

In one study, Rakel and colleagues (82) randomly assigned patients with a common cold to meet with a provider in either a standard visit (e.g., taking medical history, physical exams and diagnosis, limiting touch, eye contact, and visit time) or an enhanced visit involving setting more positive expectations about healing, expressing empathy, empowering and connecting with patients, and educating patients about their illness and treatment to a greater extent (83). The “enhanced interaction” examined in this study reduced the severity and duration of patients’ colds, and boosted IL-8 and neutrophil count. Though the researchers largely intended this interaction to bolster perceived provider empathy, many of the behaviors map onto the broader and more comprehensive dimensions of competence and warmth. For example, patients in the enhanced condition received more information about care, including written notes (relevant to competence), and experienced warmth-related non-verbal behaviors (e.g., handshakes, increased eye contact). Some manipulations may have simultaneously conveyed both warmth and competence (e.g., individualizing patient care). **Table 6** illustrates how the qualities can be organized along the competence and warmth dimensions.

**Table 6:** Experimentally varying warmth and competence in enhanced patient–provider interactions, as reported in Rakel et al. (82) and Barrett et al. (83).

	Competence	Warmth
Verbal cues	• Additional information about how to address illness • Provide written instruction on self-care • Responded to questions	• Active listening • Empathetic statements regarding condition (e.g., it’s normal to be worried, colds can sap energy) • Used humor where appropriate
**Bridging competence and warmth**		
• Included personalized comments to tailor care to individual		
		**Warmth**
Non-verbal cues		• Handshake greeting • Caring facial expressions • Increased eye contact

Another study experimentally altered patient–provider interaction in hypothetical vignettes in order to assess its relationship to malpractice claims (84), focusing on physician communication behaviors that, in pilot data, surfaced as the most important for enhancing patient–provider rapport. They essentially varied provider competence (e.g., giving information and advice) and warmth (e.g., whether they seemed judgmental and critical *vs*. warm, friendly, and attentive), as well as several components bridging competence and warmth (e.g., engaging the patient, using straightforward language) (see **Table 7**).

**Table 7:** Experimentally varying warmth and competence in enhanced patient–provider interactions in Moore et al. (84).

	Competence	Warmth
Verbal cues	• Gave patient additional information about what to expect • Gave patient additional advice/strategies	• Greeted patient warmly • Apologized for delay • Asked informal questions • Made empathetic statements (e.g., offered condolences) • Was not critical or judgmental of patient
**Bridging competence and warmth**		
• Used easy-to-understand language • Explained medical terms when used • Encouraged patient to ask questions		
	Competence	Warmth
Non-verbal cues	• Did not seem in a hurry	• Made eye contact • Listened carefully to patient

Several other studies manipulating patient–provider interactions have focused on training communication skills, as reviewed by Kelley et al. (85). These interventions have often leveraged components that can be understood using the competence and warmth framework. For example, one intervention trained physicians on several skills related to competence (e.g., repeating and summarizing important information; making referrals if needed) and several skills related to warmth (e.g., establishing rapport by introducing themselves and making eye contact; conveying empathy), as well as encouraging physicians to check patient preferences and provide information accordingly (i.e., both competence and warmth) (86, 87). Another intervention involved physicians giving more detailed explanations and making thoughtful pauses (competence) and enhanced active listening and positive non-verbal behavior (warmth), as well as developing skills relevant to competence and warmth (e.g., checking patient understanding and sharing the decision-making process) (88, 89). Yet another involved training a variety of skills that require both competence and warmth, such as assessing what the patient knows about their condition and providing information relevant to the patient’s understanding and interests (90, 91).

The methods used in these studies highlight the utility of the competence and warmth framework. In these studies, researchers often work to carefully design studies that experimentally test dozens of different components in the patient–provider interaction. Yet all of these components can be understood, categorized, and synthesized within the framework of competence and warmth. This applies across a wide variety of intervention types, including those focused on empathy, communication skills, shared decision-making, and patient-centered care.

#### Which is More Important in Patient–Provider Interactions: Competence or Warmth?

The question of whether competence or warmth is more important in social interactions has been discussed somewhat in the social perception literature. Importantly, past research suggests that warmth and competence are not necessarily a trade-off (21, 92). In fact, these dimensions often correlate somewhat positively (i.e., someone who is perceived as warmer also tends to be perceived as more competent) (17, 21).

There is some research suggesting that warmth takes primacy, or is prioritized, in judgments of others (14). When asked to list the traits that are most important in others, people tend to list warmth-related traits rather than competence-related traits, and prefer to learn about warmth-related traits in order to form impressions of others (93). Warmth judgments may also be made more quickly than competence judgments (94). Researchers suggest this pattern may occur because warmth more reliably indicates potential costs and benefits associated with interacting with another person (93, 95). Warmth’s primacy makes sense from an evolutionary perspective, as its detection separates foe from friend, potential harm from potential help (15, 94) and must be made most rapidly in order to effectively prepare to fight or flee. The primacy of warmth does not, however, indicate that it is fundamentally more important than competence; both remain essential qualities of social interactions and we propose that the same is true for patients’ interactions with providers as well.

There are differences in the role of competence and warmth in patient–provider interactions, as compared to social interactions more generally, that are worth considering. To illustrate this, consider the definitions in the social perception literature of competence as traits that are “self-profitable” (i.e., that benefit the person who possesses them), and warmth as traits that are “other-profitable” (i.e., that benefit the people around the person who possesses them) (27, 96–98). Such definitions could further justify the primacy of warmth, as they portray judgments of another person’s warmth (i.e., “Does this person possess traits that are likely to benefit me?”) as the most relevant for self-interest. But in medical care, this distinction cannot be made. A provider’s competence is clearly also “other-profitable” for patients, as its presence or absence directly affects a patient’s health outcomes. A provider needs to have their patient’s interests at heart, but without the ability to enact those positive intentions, even the best intentions are rendered meaningless. Similarly, a provider who has the knowledge to treat a patient but lacks the care or concern to thoughtfully administer this treatment will also not be effective. Accordingly, assessments of positive intentions (warmth) and the ability to enact those positive intentions (competence) are both critical in judgments of providers. Thus, a provider who seems *both* credible and likeable may be the most likely to influence patients’ health.

### Summary

Perceptions of the degree to which a provider “gets it” (i.e., competence) and “gets me” (i.e., warmth) emerge as two key dimensions in a number of important medical sources including: a) theoretical models of effective medical care, b) measures of patient satisfaction, c) measures of effective patient–provider interactions, and d) empirical research on patient–provider interactions. This suggests that the medical literature has implicitly deemed these two dimensions as pervasive and essential even if researchers did not explicitly use the terms competence and warmth. Likewise, the psychological literature has identified these same dimensions as cornerstones of impression formation more generally.

Thus, the psychological and medical literatures can be connected and simplified by utilizing the framework of competence and warmth. Competence and warmth distill a host of complex provider characteristics that are deemed essential to effective healthcare into two core dimensions. Accordingly, the competence and warmth framework can help practitioners and researchers alike identify which provider qualities are influential in patient–provider interactions and foster greater understanding of how to embody these core qualities to patients.

## Do Competence and Warmth Moderate Placebo Response?

We now turn our attention to examining whether the dimensions of competence and warmth moderate placebo response. To do so, we review four empirical studies which experimentally altered elements of patient–provider interactions to test this question (10–13).

One study deliberately manipulated competence and warmth and three of these studies (10, 12, 13) did so implicitly, although the researchers may not have explicitly set out to do so. **Table 8** illustrates how the interpersonal variables altered in these studies map onto the competence and warmth dimensions. Next, we review each of these studies and their methods in detail.

**Table 8:** Competence and warmth as dimensions of patient–provider interaction manipulations that enhanced placebo response.

Verbal cues	Competence	Warmth
Howe et al. (11)	• Articulate *vs*. filler words • Clear, confident tone *vs*. not	• Introduce self *vs*. not • Call patient by name *vs*. never ask for patient name
Czerniak et al. (12)	• Ask patient to describe pain using metaphors *vs*. don’t ask patient to describe pain at all • Emphasize has been studying pain for many years *vs*. not	• Stand to greet patient by name *vs*. remain seated Shake hand and invite in *vs*. not • Repeat patients’ answers to questions *vs*. merely type answer
Kaptchuk et al. (10)	• Ask additional questions about symptoms *vs*. state had reviewed questionnaire • States that have had much experience with treatment *vs*. not	• Warm/friendly manner *vs*. state cannot converse with patients • Use empathetic statements *vs*. not • Actively listen (e.g., repeat patient words) *vs*. not • Words of encouragement *vs*. not
Fuentes et al. (13)	• Ask additional questions about patient symptoms	• Actively listen (e.g., repeat patient words) *vs*. not
		• Use empathetic statements *vs*. not
		• Words of encouragement *vs*. not
**Bridging competence and warmth**		
• Ask patient how they normally address pain *vs*. not (Czerniak) • Ask additional questions about patient understanding of condition *vs*. not (Kaptchuk) • Ask additional questions about conditions’ impact on life *vs*. not (Kaptchuk) • Ask about patient lifestyle (Fuentes) • Ask about patient understanding of causes of symptoms (Fuentes)		
Non-verbal cues	Competence	Warmth
Howe et al. (11)	• Make no mistakes *vs*. putting blood pressure cuff on upside down	• Make eye contact *vs*. stare at computer • Sit closer to patient *vs*. maintain physical distance
Czerniak et al. (12)	• Look at cell phone *vs*. not interrupted by cell phone • Examine patient closely *vs*. briefly and more distantly • Hand cream to patient with a large gesture *vs*. not	• Make eye contact *vs*. stare at computer • Patient inspection involves touch *vs*. patient inspection is only visual
Kaptchuk et al. (10)	• 20 seconds of thoughtful silence during procedure *vs*. not	
Fuentes et al. (13)		• Use of physical touch *vs*. not • Increased eye contact • Warm tone of voice
Environmental cues	Competence	Warmth
Howe et al. (11)	• Nametag indicates higher status (i.e., MD) *vs*. lower status (i.e., student doctor) • Professional attire (e.g., long white coat) *vs*. casual, wrinkled clothes, shorter white coat • Room organized, neat *vs*. disorganized, scattered papers	• Posters with warm images (e.g., red panda) *vs*. no personalized posters
Czerniak et al. (12)	• Carefully select cream from jars *vs*. pull from top drawer of desk	

N = number of participants in the study.

### Czerniak et al. (12): Competence and Warmth Moderate Placebo Pain Relief

Czerniak and colleagues (12) found that warm and competent patient–provider interactions increased healthy volunteers’ responses to a placebo cream described as an analgesic (*N* = 122). This ostensible analgesic was applied before patients underwent a cold pressor task (99) in which participants immerse their hand in an ice water bath to induce pain. First, all participants underwent the cold pressor task without the administration of placebo cream to assess baseline pain threshold (defined as the number of seconds before participants indicated that they felt pain from the cold) and pain tolerance (defined as the number of seconds before participants withdrew their hand from the cold). Then, a trained actor posing as a doctor administered a placebo cream (i.e., moisturizer lotion) described as a pain relief cream before participants repeated the cold pressor task. The researchers randomly assigned patients to receive this placebo cream either in the context of a standard interaction designed to mimic a routine doctor’s visit, or in the context of an enhanced interaction involving characteristics of ritual healing. Both the standard and enhanced interactions lasted approximately 5 minutes or less. Placebo response was measured by pain threshold and pain tolerance relative to baseline.

The researchers drew their inspiration for the “enhanced” interaction from a shaman’s healing ritual, incorporating performance behaviors. The authors used a variety of performance-relevant behaviors in the enhanced interactions, including verbal behaviors (i.e., dialogue) that was “personal, attentive to the volunteer, and used imagery in the questions and explanations” (12, p. 4), and deliberate non-verbal behaviors, such as dramatic gestures and movement in the room. The dimensions altered, however, can be organized under the simplifying and unifying framework of provider competence and warmth. Some verbal behaviors (e.g., emphasizing that the provider has many years of experience studying pain, helping patients to use metaphors to describe their pain) and non-verbal behaviors (e.g., examining participants’ hands more closely, not being distracted by a cell phone during the interaction) likely increased perceived competence. Several other verbal behaviors (e.g., greeting the participant by name) and non-verbal behaviors (e.g., increasing eye contact, using physical touch) likely increased perceived warmth. Some manipulations may have targeted both competence and warmth. In the enhanced interaction, the provider asked the patient to describe how they normally treat pain, thereby taking the patient’s own preferences into account (signaling warmth) and gathering additional information to shape treatment decisions (signaling competence).

Participants who experienced the “enhanced” interaction showed a higher pain tolerance during the cold pressor task compared to participants who experienced the standard interaction. However, the effect of the interaction on pain tolerance was limited to participants who were categorized as “placebo responders” (defined as participants who showed at least a 30% increase in pain tolerance after placebo administration), suggesting that participants who were not susceptible to placebos were also not influenced by the differences in provider interactions.

### Kaptchuk et al. (10): Competence and Warmth Moderate Placebo Treatment for IBS

Kaptchuk et al. (10) found that warm and competent patient–provider interactions increased patients’ response to sham acupuncture administered over the course of 3 weeks to treat irritable bowel syndrome (IBS) (*N* = 262). Sham acupuncture uses a device that creates the appearance of having pierced the skin without actually doing so, in order to mimic the needles used during acupuncture. Patients were randomly assigned to either receive this sham acupuncture in a short interaction in which providers restricted their engagement with patients, or in an enhanced interaction in which providers engaged in additional conversation with patients and incorporated several verbal and non-verbal behaviors to improve the quality of the interaction. Placebo response was measured through self-reported improvement in IBS symptoms, self-reported adequate relief of IBS symptoms, self-reported symptom severity, and the self-reported degree to which the condition interfered with a patient’s quality of life.

The enhanced interaction in this study (10) was designed to be “warm, empathetic, and confident” (p. 2), clearly covering the two dimensions of provider competence and warmth. As documented in **Table 5**, several verbal behaviors (e.g., stating that the provider has had much experience with the treatment) and non-verbal behaviors (e.g., pausing in thoughtful silence for 20 s during the procedure) may have evoked competence, and several verbal behaviors (e.g., making empathetic statements, using active listening and words of encouragement) may have evoked warmth, and some behaviors may have evoked both competence and warmth (e.g., asking additional questions about the patient’s understanding of the treatment).

Patients who experienced the “enhanced” interaction reported greater relief and improvement in symptoms over the course of the 6-week study. Thus, the positive effects of placebo acupuncture were augmented by a more supportive interaction with a provider.

### Fuentes et al. (13): Competence and Warmth Moderate Placebo Treatment for Chronic Low Back Pain

Fuentes et al. (13) used a similar protocol to Kaptchuk et al. (10) to enhance the interaction between therapists and patients with chronic low back pain who were randomly assigned to either undergo active interferential current therapy (IFC) or sham IFC (*N* = 117).

In one condition, patients experienced a limited interaction in which the provider left after briefly introducing themselves and explaining the treatment. Providers also mentioned that they had been instructed not to converse with participants and minimized discussion accordingly. In the “enhanced interaction” condition, patients experienced an enhanced interaction involving several verbal behaviors that may have enhanced perceived competence (e.g., the provider asked patients additional questions about their symptoms), several that may have enhanced perceived warmth (e.g., active listening, making empathetic statements such as “I can understand how difficult this must be for you”), and several that may have targeted both (e.g., asking patients about their lifestyle and assessing their understanding of their condition). Enhanced interactions also employed several non-verbal behaviors that conveyed warmth, including a warmer tone of voice, increased eye contact, and incorporating physical touch into treatment.

The authors found that the enhanced interaction improved outcomes for both active and placebo treatment. As with Kaptchuk et al. (10), the enhanced interaction also involved providers spending more time with patients (5 min in the limited interaction and about 30 min in the enhanced interaction).

### Howe et al. (11): Competence and Warmth Moderate Placebo Treatment for Allergic Reactions

The only study to date which has altered provider warmth and competence *independently* from each other in order to tease apart the dimensions was done by Howe and colleagues (11). In this study, healthy volunteers (*N* = 164) underwent a skin prick test using histamine, which was administered by a trained research assistant who acted as the provider. (Histamine causes a mild allergic reaction in which the skin becomes red, itchy, and a small bump called a “wheal” surfaces.) The provider then applied a placebo cream (moisturizer lotion) to the allergic reaction. This study also separated the qualities of the interaction from the expectations set about the placebo treatment. In the positive expectations condition, they stated that the cream was an antihistamine cream that would reduce the reaction and decrease itching. In the negative expectations condition, they stated that the cream was a histamine agonist that would increase the reaction and increase itching. Placebo/nocebo response was measured by the change in participants’ wheal size (in mm) after the placebo cream was applied.

The same provider administered the cream to all participants, but was trained to interact with participants in one of four ways to evoke: 1. High warmth and high competence, 2. High warmth and low competence, 3. Low warmth and high competence, or 4. Low warmth and low competence. Competence was evoked through verbal manipulations (e.g., speaking confidently, minimizing filler words), non-verbal manipulations (e.g., executing all procedures flawlessly), and environmental manipulations (e.g., professional attire, room neat and clean). Warmth was also evoked through verbal manipulations (e.g., the provider introducing themselves and calling the participant by name), non-verbal manipulations (e.g., increased eye contact, sitting closer to participant), and environmental manipulations (e.g., hanging posters with warm images in the exam room). All conditions were the same length of time, thereby controlling for time interacting with the provider. Patients’ self-reported ratings of the provider at the end of the exam suggested that perceived competence and warmth were substantially impacted through these simple changes, suggesting that perceptions of providers’ warmth and competence are readily malleable.^3^

The researchers found that competence and warmth moderated placebo and nocebo responses. When the provider appeared both competent and warm, participants who heard positive expectations about the cream showed a greater decrease in wheal size than participants who heard negative expectations about the cream. However, when participants had interacted with a provider who was low in warmth and low in competence, their wheal size continued to increase at the same rate regardless of whether or not the provider had set positive or negative expectations about the cream. Mixed conditions (i.e., high warmth/low competence and low warmth/high competence) produced moderate effects on the allergic reaction and were indistinguishable from each other.

This study disentangled precise dimensions of patient–provider relationships and found that warmth and competence shape participants’ physiological responses to the expectations that a provider sets about treatment. An additional important take-away from this study is that neither warmth nor competence seemed to matter more than the other; rather, it was only when the two qualities worked together that they effectively created an overall interaction that boosted placebo effects.

### Summary

Overall, these studies support the notion that a provider’s competence and warmth are key dimensions that moderate placebo response: interactions in which a provider demonstrated both competence and warmth resulted in a greater response to placebo and active treatments. Thus, whether a provider “gets it” and “gets me” can affect the potency of a medical treatment. Accordingly, both of these dimensions constitute an important part of effective healthcare.

## What Are the Mechanism Through Which Competence and Warmth Moderate Placebo Response?

The patient–provider relationship is frequently cited as a key mechanism of placebo effects in and of itself (10, 83, 85). As discussed in depth above, the patient–provider relationship assessed in placebo research clearly contains dimensions of both competence and warmth. However, the mechanisms through which a competent and warm patient–provider interaction might boost placebo response are unclear from past literature. We propose that provider competence and warmth increases overall placebo effects by boosting known placebo mechanisms, including a) expectations and b) classical conditioning (i.e., repeated associations between a medical stimulus, such as a pill, and the active drug inside the pill, which could lead to a conditioned response) (4, 100). By augmenting the impact of these known placebo mechanisms, provider warmth and competence then boost overall placebo response.

### Competence and Warmth Amplify Patient Expectations About Treatment

A provider’s competence and warmth make a provider more credible, believable, and/or persuasive (101), which may boost the impact of the expectations they set about treatment. A doctor who is competent (e.g., conducts a thorough exam, seems knowledgeable) will appear as a more reputable source of medical information. Thus, the patient may be more likely to internalize this competent doctor’s message about a treatment’s efficacy. Likewise, when a doctor is warm (e.g., is friendly, calls the patient by name), the patient may feel more relaxed, at ease, and like they are in good hands. The patient may then be more receptive to what the doctor has to say, view the doctor as trustworthy, and believe expectations set about the efficacy of treatment to a greater extent. A warm provider may also appear to better understand the patient, and thus enhance this patient’s confidence that the provider has chosen a course of treatment that will work for them as an individual. Patients may thus listen to and trust explanations of warm and competent providers to a greater degree, and accordingly be more influenced by them physiologically (102–104).

Competent and warm providers may thus be better able to set specific, individualized expectations that are more meaningful, helpful, and relevant for patients. When expectations resonate with patients more, they increase healing to a greater degree (105). Similarly, competent and warm providers may also more effectively set expectations about patients’ own role in their health management. For example, one study examining enhanced provider interactions included provider comments such as “You can really make a difference in your cold by taking care of yourself” (82, 83). Such a statement may have no potency if a provider seems to lack understanding of medicine and/or of a particular patient’s needs and abilities, but may be particularly believable coming from a provider who is seen as competent and warm. As another example, warm and competent providers may also be more skilled at reassuring patients in the course of treatment by providing information clearly and confidently, and providing concern that seems authentic. This could positively impact patient expectations by, for example, resolving uncertainty (106, 107). Furthermore, a recent study shows that even without medication, physician reassurance can help patients feel better by reducing symptoms and speeding healing (108). Through such processes, competent and warm providers may more effectively leverage the healing that is evoked by setting patients’ expectations about treatment.

### Competence and Warmth Activate Conditioned Patient Responses

Competent and warm providers may more effectively leverage strategies that boost conditioned responses (109), including diagnostic rituals such as the physical exam. Further, competent and warm providers may simply *feel* more like a healer to the patient, thus leading the patient to experience greater conditioned responses. We thus theorize that warm and competent providers may activate conditioned patient responses because they are more effective at engaging in healing rituals that produce conditioned responses, and because patients may experience a greater conditioned response to these providers themselves.

It has been widely acknowledged that healing rituals can lead to conditioned placebo responses (10, 12, 100, 110). Even normal, everyday procedures that rely on only basic medical competence, such as taking a patient’s height, weight, and blood pressure, can become conditioned stimuli for healing in a clinical context (105). However, there is likely great variation in how effectively different providers utilize healing rituals. Warmer, more competent providers may more effectively engage in rituals that produce conditioned healing responses in patients. For example, the physical exam may not only lead to more and better information with which to heal patients, it is likely that the “laying of hands” in the physical exam is healing in and of itself (111–113). Likewise, research on the meaning of touch for patients with cancer found that nurses’ touch conveyed confidence to these patients, and this confidence in turn increased positive patient expectations and hope of recovery (114). But touch can also be aversive for some patients—if a provider is not warm and competent, then these rituals could backfire. Providers who are competent and warm—who are socially and emotionally skilled and able to quickly gauge what their patients prefer—may be better able to utilize medical rituals effectively, particularly rituals involving touch. Indeed, provider warmth and competence may be crucial in the success of these rituals, as these dimensions may be the difference between a ritualistic experience that boosts healing and one that is off-putting for the patient.

Research also supports the hypothesis that a competent and warm provider may activate or amplify conditioned patient responses. Some research suggests that providers who seemed more like an expert or fit certain stereotypes about a doctor were able to enhance response to a treatment regardless of whether they used a placebo or active acupuncture treatment (115). Providers who are competent and warm may thus seem more like a good doctor or a trustworthy expert, which could bolster a conditioned response to seeing such a provider. While participants in past research have been shown to display conditioned responses to doctors who better fit stereotypical images of doctors (i.e., White male doctors), as medicine grows ever-more diverse, aspects of the provider, such as warmth and competence, may rise up in place of physical attributes to produce conditioned responses in patients. We are not aware of any research that directly assesses the impact of provider competence and warmth on conditioning, and future research should investigate how qualities of the provider may amplify or otherwise influence the effects of conditioned healing.

### Summary

We have proposed that competence and warmth play a key role in placebo effects by strengthening expectations and conditioning during medical treatment. Of course, being complex psychological phenomena, provider competence and warmth likely impact placebo response in many other ways, including by reducing stress and anxiety, increasing positive emotions, influencing physiology directly, and by beneficially impacting behavioral mechanisms such as adherence, motivation, and adoption of healthier behaviors (82, 83, 101, 116–123). Indeed, past research and theory have suggested that provider competence and warmth can set off a cascade of physiological changes in the body, including “endogenous neurotransmitters, hormones, and immune regulators that mimic the expected or conditioned pharmacological effects” (124). But given the known importance of expectations and conditioning for placebo effects and the attention paid to these mechanisms in the placebo literature (3), we have restricted our discussion to these mechanisms and encourage future research and theory on other mechanisms.

## How Can Providers Deliberately Leverage Competence and Warmth in Clinical Care?

In order to leverage competence and warmth in healthcare, we need to first understand what these qualities look like from a patient perspective and how they might reasonably be enacted from a provider perspective. To this end, we asked both patients and providers to describe their healthcare experiences. Their responses capture patients’ and providers’ impressions of how competence and warmth can be demonstrated in clinical encounters.

### Provider Competence and Warmth From a Patient Perspective

To find out what provider competence and warmth look like to patients and how providers might embody this in real-world settings, we asked participants to describe healthcare experiences in open-ended responses.

Participants first answered two questions in which they imagined what positive qualities and behaviors a good doctor would demonstrate:

1. Imagine what a good doctor would be like. What good things would this doctor do?
2. What good qualities would this doctor have?

Then, participants reflected on their own experiences. Participants first responded yes or no to whether they had ever seen a good doctor, and yes or no to whether they had ever seen a bad doctor. If respondents answered yes to one or both questions, they were asked, respectively:

3. What was good about this doctor? and/or
4. What was bad about this doctor?

These questions allowed us to assess qualities and actions drawn from both patients’ own positive or negative interactions with providers and patients’ ideal interactions with providers.

In total, 334 American participants between age 25 and 87 (51.2% women, *M*_age_ = 43.10, *SD*_age_ = 14.09) responded to the survey, which was administered by Survey Sampling International (SSI). Participants came from a variety of racial/ethnic backgrounds [29.6% White/Caucasian, 24.9% Asian/Pacific Islander, 23.4% Black/African-American, 22.2% Hispanic/Latino (a)] and socioeconomic backgrounds (41.0% college education, 28.8% some college education, 21.0% high school or less). Detailed survey methods are described in previous publications (125).

Following similar procedures to previous research (125), the authors generated a coding scheme including five categories related to a provider’s competence and four categories related to a provider’s warmth (see **Table 9** for a description and examples of each category).

**Table 9:** Competence and warmth demonstrations and examples from patients.

Category		
**Competence/“gets it”:** *Related to a provider’s effectiveness at diagnosing and treating disease/symptoms of disease and encouraging healthy habits*		
**Subcategory**	**Description**	**Examples**
“Medically knowledgeable” (general knowledge)	The doctor is medically knowledgeable, knows current research and practices, intelligent, well-educated.	What good qualities would this doctor have? “Good education” “Up to date with newer medical studies” What good things would this doctor do? “Be smart” “Keep me well informed about newest developments” What was good about this doctor? “He had a good knowledge of his field.” “Gives proper treatment” What was bad about this doctor? “Incompetent” “Could not explain the importance of a balanced nutrition”
“Keeps at it” (thoroughness)	The doctor has an attention to detail, is thorough, covers all alternatives, has a good work ethic.	What good qualities would this doctor have? “Looks at any and all alternatives” “Would honestly do everything he can to help me” What good things would this doctor do? “Check me out thoroughly” “Would follow-up on small concerns” What was good about this doctor? “Attention to detail” “She was thorough.” What was bad about this doctor? “Very rushed” “Not interested in your illness, just what prescriptions do you need”
“Understands my health” (patient-specific medical knowledge)	The doctor knows your health history, has experience with patients like you (e.g., demographically, or with particular conditions).	What good qualities would this doctor have? “Experience treating similar people” “Know my medical record for all appointments” What good things would this doctor do? “Know your body, habits, and family history” “Personalized patient care” What was good about this doctor? “Knows about my health” “Knew our family history” What was bad about this doctor? “Never had a patient who exhibited similar symptoms” “Ignoring available information about my history”
“Has seen it” (experience)	The doctor has a lot and/or a variety of medical experience, has been practicing medicine for many years, has seen a lot of patients and treated a lot of medical conditions generally, knows their skill set/limitations.	What good qualities would this doctor have? “Very experienced” “Not attempt any treatment beyond that which he is skilled” What good things would this doctor do? “Know the area of his practice” “Refers you to specialist as needed What was good about this doctor? “He knows what he’s talking about.” “If can’t help, finds someone who can”
“Walks the walk” (role modeling)	The doctor maintains their own physical and mental health.	What good qualities would this doctor have? “Practices a healthy lifestyle themselves” “A great role model” What good things would this doctor do? “Eat healthy” What was good about this doctor? “Practiced what he preached”
**Warmth/“gets me”:** *Related to a provider’ viewing and/or treating the patient as a social being, including acknowledging their perspective about life and/or health*		
“Is nice to me” (general warmth)	The doctor is friendly, open, caring, empathetic, respectful, has people skills.	What good qualities would this doctor have? “Care about what is wrong with you” “A warm smile” What good things would this doctor do? “Have empathy” “Not get irritated or mean when I need help” What was good about this doctor? “Was very nice” “Had genuine concern for my well-being” What was bad about this doctor? “Disrespectful and rude” “Lack of compassion”
“Hears me” (active listening)	The doctor has good interpersonal skills, listens carefully, makes patient feel at ease, treats patient as an equal.	What good qualities would this doctor have? “Listens to your concerns” “Easy to talk to” What good things would this doctor do? “Talk to me like I am an intelligent person” “He listens and believes me” What was good about this doctor? “Good people skills and great communicator” “Listened to what I was saying” What was bad about this doctor? “Didn’t talk to me” “Inability to listen”
“In it for the right reasons” (passion for people)	The doctor practices medicine to help people, loves what they do.	What good qualities would this doctor have? “A people person who really cares about people” “Passionate about what they do” What was good about this doctor? “Good heart for the people” “Loved their job” What was bad about this doctor? “Was not humanitarian”
“Takes the patient’s perspective” (patient-specific warmth)	The doctor knows who the patient is and treats them as an individual, understands the patient’s personal life, background, culture, worries, values, etc., thinks about a patient’s individual goals, needs, and perspectives.	What good qualities would this doctor have? “Do not treat you just as a patient but as a person” “Interested in me as an individual” What good things would this doctor do? “Try to relate to me on a personal level so I feel comfortable with any diagnosis they give” “Cares about you as a person” What was good about this doctor? “Asked questions about what was going on in my life” “Treated me as a person, not an illness” What was bad about this doctor? “Didn’t take the time to get to know me” “Didn’t bother treating me like a human”

Two research assistants who were blind to hypotheses coded a randomly selected 20% of participant responses (*N* = 67 each) by indicating whether participants mentioned this category (1) or did not mention this category (0) for each of the four questions. Coders first coded 20% of the responses and then discussed and reconciled any discrepancies before coding the 80% of responses (*N* = 100 each). Inter-rater agreement before coders began coding the full sample was acceptable (Cohen’s kappas > 0.70 for all categories). Data and scripts for analysis are provided at https://osf.io/5jxqy/.

**Table 9** depicts the different ways patients have experienced various forms of competence and warmth in their interactions with providers. These data illustrate that there is a rich variety of ways in which providers can demonstrate competence and warmth to their patients. Of course, providers do not need to embody all of these qualities or perform all of these actions. **Table 9** is not meant to be a checklist for effective medical care, but rather a rolodex of possible tools providers could employ to bolster competence and warmth. Ultimately, what appears to matter for healthcare is that patients perceive a provider as “getting it” and “getting me,” and there are many routes to these same ends.

### Competence and Warmth in Providers’ Own Words

In addition to patient perspectives, we turned to medical providers to understand what competence and warmth actually look like in clinical practice. During focus groups in four Primary Care clinics, care team members were asked to generate ways they signal competence and warmth to patients. We collected responses from approximately 100 care team members, including physicians, medical assistants, nurses, and clinic staff.

Responses were collected during a larger training session, which also explained competence and warmth in the “gets it” and “gets me” framework. Providers were then asked: “How do you signal to patients that you get both ‘it’ and ‘them’?” Providers listed at least one example of how they signal competence to patients (getting “it”) and at least one example of how they signal warmth to patients (getting “them”). Providers’ responses were coded and grouped into thematically similar strategies. **Table 10** lists the overarching strategies that emerged from providers’ responses, and displays exemplary quotes for each category in providers’ own words.

**Table 10:** Competence and warmth strategies and examples from the healthcare team.

Category		
**Competence/“gets it”:** Related to a provider’s* effectiveness at diagnosing and treating disease/symptoms of disease; a provider’s understanding of diagnosis, prognosis, and treatment		
**Strategy**	**Description**	**Examples**
“Review ahead of time”	The provider reviews relevant information on the patient, potential options, procedures, and treatments before the medical encounter in order to prepare.	Review medically-relevant information “Make sure you are familiar with the topics you discuss” “Have a couple of options in mind for therapy/plan before appointment” Review patient condition “Briefly reviewing chart before visit so patients feel that you are updated with their recent health changes/specialist views” “Not asking questions easily seen by chart review” “Reference things in the patient’s chart to demonstrate that I’ve familiarized myself with their case”
“Encourages questions”	The provider encourages the patient to ask questions and have questions answered.	“State that you understand & address questions about their disease/treatment” “Ask if and answer any questions the patient has”
“Provide explanations”	The provider, upon hearing the questions and concerns of the patient, provides clear and informative answers.	“Very informational” “Explaining carefully”
“Share helpful information”	The provider shares information to inform the patient on their own role and experience, on the medical conditions and procedures, and on the medical institution. The patient thus feels well-informed and part of the decision-making process.	Share about the provider’s role and experience “Relate their situation to another similar situation I’ve worked on and resolved” “Say, ‘I’ve seen this many times’” “Explain my role and how I can help them” Share medically-relevant information “Explain rationale for treatment, including previous patient experiences” “Explain procedures in detail with examples” “When going over I advise patient on appropriate time frames for things they are due for and the importance of health maintenance.” “Pull up supporting evidence; online resources to help guide our decisions” Share about medical institution “Knowledge of the hospital success measurements” “Use (name of institution) and our excellence, resources for context”
“Personalize medical care”	The provider understands the unique context and history of the patient, catering to their specific needs when providing medical care.	“Say, ‘There are lots of ways to achieve improvement, and I/we want to find one(s) that fit you best’” “Say, ‘“As part of your (name of clinic) care team, we are looking for a personalized approach for you’”
“Be transparent”	The provider demonstrates honesty and transparency when faced with questions they do not have the answers to. They show a commitment to learning the answers and following up with the patient.	“If you don’t know how to address something, instead of making something up, validate and give direction” “I admit when I’m not sure or don’t know and offer continued research or collaboration with MD” “Being open about areas of ambiguity in outcomes/diagnoses/management/follow-up plan”
“Show confidence”	The provider conveys confidence in their role, work, experience, and surrounding environment.	“Provide statements with confidence, meaning not to be too wishy-washy about it” “Have my routine and comfortably complete it; confident in my environment” “Confidence in delivery of assessment & preparation for it”
“Have familiarity with procedures”	The provider is prepared and comfortable with the medical procedures performed.	“Keep arm elevated during blood pressure” “How relaxed you are while performing a procedure” “Take their weight, height, vitals” “Do procedures calmly/don’t seem flustered”
“Appear presentable”	The provider presents themselves and their environment as being put-together.	“Wear professional clothes” “Keep the room clean” “Organized exam room for regular visits & for procedures”
“Collaborate well with team”	The provider holds an understanding of how they are one piece of a medical care team, leveraging their team members to contribute to the same goals.	“Everyone giving the same message & on the same page” “Say, ‘I’m part of a team of ___ here to serve you and be sure you get the best care’” “Collaboration with peers” “Say, ‘Dr. __ is the best!’”
**Warmth/“gets me”:** *Related to a provider viewing and/or treating the patient as a social being, including acknowledging their perspective about life and/or health; a provider’s understanding about the patient’s goals, needs, and concerns*		
**Strategy**	**Description**	**Examples**
“Greet effectively”	The provider understands the importance of starting the encounter off on the right foot to make patients feel valued.	Introduction of provider “Introducing myself at beginning of visit” Communicate through body language “Greeting the patient with a smile and a handshake” “Nonverbal cues—eye contact, smile—when opening the encounter” “Open the door for them” “Ask, ‘what brings you in to see your doctor?’ with eye contact” Use patient’s name “Always greet by preferred name and gender” “Call them by their first name with a huge smile on my face”
“Use intentional body language”	The provider’s body language and nonverbal cues communicate that the patient is the focus of attention.	“Always smiling at patient” “During the first few minutes of connecting, make eye contact with patient and not computer” “Empathetic touch when upset” “Sit down when talking to patient”
“Form personal connection with patient”	The provider cares for the patient as a whole person, showing curiosity for and investment into their lives beyond the context of the medical encounter.	“Opening and closing each visit with brief small talk” “Ask about holiday plans and family details” “Say, ‘I’m so glad to see you today’” “Establish rapport by asking personal questions” “Judicious sharing of personal information”
“Remember past details about patient”	The provider forms a relationship with the patient over the long-term, thoughtfully referencing previous encounters and details.	“Know something of major significance about their lives (e.g., going through a divorce” “Talk about a previous subject discussed during last appointment” “Say name of patient, family members, pets”
“Value patient’s needs”	The provider prioritizes the patient’s needs, concerns, and perspective. They are able to give space for the patient to express themselves and then respond to demonstrate that the patient has been heard.	Listen “Actively listening to their concerns” “Listen generously; use silence” “Repeat my understanding of what patient says” Be attuned to patient’s needs “Know their health care goals” “Ask them what their thoughts are about the proposed treatment” “Anticipate patient’s need by asking before leaving room”
“Convey empathy”	The provider empathizes with where the patient is coming from and conveys that they are alongside the patient as support.	“Regardless of their reason for visit, I always tell the patient, ‘Don’t worry, we will take care of you today!’” “Use ‘we’ to show that we are in this together” “Validate their feelings and expressions of concern” “Use cultural intelligence and respect”

*For this table, “providers” refers to the entire care team at several Primary Care clinics, and thus includes physicians, medical assistants, nurse practitioners, front desk staff, behavioral health specialists, and pharmacists.

Importantly, as with **Table 9**, **Table 10** is not meant to suggest that providers adopt all of these strategies. Rather, **Table 10** suggests a multitude of ways in which providers could bolster patient perceptions of competence and warmth, allowing providers to flexibly choose strategies that resonate with them and/or their patients’ needs. Providers’ responses span a wide range of behaviors, suggesting that everyone on the care team can bring their own unique strengths to signaling competence and warmth in clinical encounters. Critically, since these responses were generated from all members of the care team, they encompass ways each person in a healthcare clinic could signal competence and warmth to patients, whether their role is as a physician interacting with patients intimately or a scheduler who only interacts with patients by phone. Providers can thus take away from these responses what is most useful and actionable for them given the particular demands and resources of their healthcare context.

While some of these behaviors are basic, intuitive practices (e.g., eye contact), others require the cooperation of multiple medical team members (e.g., consistent messaging to patients). Some require greater investments of time and effort, such as researching personalized treatments beforehand and asking patients about their concerns. However, there are also many strategies that require only intention, not additional time, such as calling patients by name, greeting them warmly, and projecting confidence. Further, even the more effort-intensive demonstrations of competence and warmth may save providers more time in the long-term by fully addressing patients’ needs.

## Summary and Future Directions

By framing patient–provider interactions in terms of provider competence and warmth, we have capitalized on decades of research in social perception to begin to unpack how and why patient–provider interactions can boost placebo response. We have also begun to identify ways providers can leverage competence and warmth to deliberately increase the strength of placebo response. The competence/warmth framework simplifies the complex patient–provider interaction, organizing dozens of behaviors and qualities into two key dimensions that can be bolstered through a variety of routes. It thus suggests to clinicians and researchers alike what to focus on to enhance patient–provider interaction quality and suggests many practical ways to leverage the power of the patient–provider relationship to boost placebo effects. It is our hope that the framework of competence and warmth will provide researchers and practitioners alike with a theoretical grounding from which to understand what aspects of the patient–provider interaction are most critical for improving various outcomes of medical care.

Further, we have illustrated how this framework is present in both placebo and medical literature, as evident in the way studies alter patient–provider interactions and how patient–provider interactions are assessed. This framework thus unites literature on social perception, placebo research, and medical research. In addition, considering the influence of competence and warmth could help generate novel ideas about the mechanisms through which patient–provider interactions may boost placebo effects. We have proposed that competence and warmth make a provider seem more credible and foster patients’ belief in them and their statements, and thus enhance the impact of treatment expectations. We have also proposed that a provider’s competence and warmth strengthen conditioned responses to providers and to medical rituals. There are a variety of other possible mechanisms through which a provider’s competence and warmth may influence placebo effects and patient health more broadly (e.g., reducing anxiety).

It is likely that the qualities of competence and warmth foster other benefits in patient–provider interactions beyond enhancing patients’ placebo response. For example, a provider’s competence and warmth may establish trust between patients and providers. Indeed, competence and warmth emerge as core dimensions in literature on the social perception of trust (126, 127). Prerequisites of trust include *ability*, or “skills, competencies, and characteristics that enable a party to have influence within some specific domain,” and *benevolence*, or “the extent to which a trustee is believed to want to do good to the trustor, aside from an egocentric profit motive” (127), dimensions that also map onto the competence/warmth framework. The possible relationship between competence, warmth, and trust in the healthcare context should be explored. Focusing on showcasing competence and warmth to patients could offer providers a more tangible route through which to establish trust than abstract recommendations to “get patients to trust you.” Demonstrations of competence and warmth may be especially important for building trust in cross-race, cross-gender, and cross-socioecomonic status interactions, where trust may be absent or more challenging to build.

The guiding framework of competence and warmth inspires many open questions and serves as a guide for future research. One question is the degree to which competence and warmth are separable in medicine. A recent study found that behaviors often used to cultivate perceptions of warmth (e.g., eye contact) bolstered perceptions of *both* warmth and competence (128). In a medical context, perhaps especially when patients are anxious about very personal concerns, “getting me” may be critical to whether a provider seems to “get it.” Likewise, the degree to which signals of warmth and competence *via* verbal, *vs*. non-verbal, *vs*. environmental cues evoke perceptions of these qualities is an open question. In addition, the universality of different experimental manipulations of warmth and competence is uncertain. For example, Kraft-Todd and colleagues (128) found that a provider wearing a white coat did not enhance perceptions of their competence; indeed, evidence on whether professional attire affects perceptions of competence is largely mixed (129–132). Another interesting question for future research is whether the impact of more general qualities of warmth (e.g., general friendliness, eye contact) and competence (e.g., general medical knowledge, articulateness) differs from the impact from patient-specific qualities of warmth (e.g., asking a patient questions about their personal life) and competence (e.g., demonstrating knowledge of a patient’s family history) (see examples in **Table 1**).

While we have proposed that warmth and competence work in conjunction to promote healing, certain contexts, patients, and circumstances may render either warmth or competence more impactful. Cultural expectations and individual personalities or desires likely play a role in both whether patients value warmth or competence more as well as how patients prefer their providers to express warmth and competence (133–135). For example, some of the behaviors patients and providers associated with warmth reviewed in this paper (e.g., calling a patient by their first name) may backfire in other cultural contexts. Different medical problems may also lend themselves more to warmth or competence; warmth might be especially important when dealing with a chronic illness that needs to be managed over time, while competence may be seen as more critical during surgery and for setting broken bones (136).

Regarding questions about the role of patient–provider relationships in placebo effects, the greatest need seems to be for rigorous research that separates the impact of provider interaction style (i.e., providers who are competent and/or warm) from the impact of explicitly set positive expectations. Future studies could help unpack whether and how provider competence and warmth boost the impact of expectations, as well as how setting expectations might boost patient perceptions of provider warmth and competence. This article hypothesizes mechanisms for how provider warmth and competence can boost placebo response, but future empirical research is needed to assess the validity of these hypotheses in research and clinical practice.

We hope that understanding and leveraging the competence and warmth framework will allow us to better address some of the most pressing problems in healthcare. For example, a wealth of literature suggests that minority populations in the U.S. have worse health outcomes (137). Recent authors suggest that differences in placebo response may be at least partially responsible for some of these disparities (138). Deliberately and effectively leveraging warmth and competence could potentially help healthcare providers diminish these gaps. Particularly as research suggests that cultural or racial matches between providers and patients lead to improved healthcare outcomes, warmth and competence may be one way to bridge the divide between providers and patients of different cultural, racial, and socioeconomic backgrounds, as it remains unfeasible to ensure that each patient is seen by a provider who matches his or her cultural background (135). Future research could explore these exciting possibilities.

It is our hope that the theory outlined in this article will spur novel research in these areas. Understanding how, when, and why provider qualities such as warmth and competence boost placebo response will not only further our comprehension of placebo effects, but will also help the medical field deliberately harness important mechanisms of placebo response that can be taken advantage of ethically alongside active medication and treatment. By distilling the complex qualities and behaviors of effective healthcare providers into warmth and competence, we hope this framework can help researchers and practitioners alike to more clearly understand how to practically and purposefully leverage the patient–provider relationship to boost placebo effects and improve healing.

## Ethics Statement

This study was carried out in accordance with the recommendations of the Stanford University Institutional Review Board. The protocol was approved by the Stanford University Institutional Review Board. The Stanford University Institutional Review Board waived the need for written informed consent from participants.

## Author Contributions

LH and KL analyzed the data and drafted the manuscript and AC provided critical revisions. All authors listed have made a substantial, direct, and intellectual contribution to the work, and approved it for publication.

## Funding

AC is supported by NIH/NCCIH Grant #DP2AT009511. AC and LH are supported by a grant from the Robert Wood Johnson Foundation. KL holds a Stanford Interdisciplinary Graduate Fellowship—Anonymous Donor.

## Conflict of Interest Statement

The authors declare that the research was conducted in the absence of any commercial or financial relationships that could be construed as a potential conflict of interest.

## Footnotes

1 For example, the dimensions of warmth and competence also model people’s judgments of the characteristics of social groups. Ratings of warmth and competence distinguished a variety of different social groups on the basis of out-group members’ stereotypes about these groups (24). Stereotypes of groups could be categorized into four unique clusters: those rated high on warmth and competence, low on warmth and competence, high on warmth but low on competence, and low on competence but high on warmth.

2 A large literature has explored provider empathy in patient-provider interactions and suggests that it can play an important role (e.g., improving patient health outcomes) (37–39). Empathy is a multifaceted construct that may include several different components, including awareness and sharing of others’ affect, caring for others’ welfare, and/or imagining what others are feeling (39). The literature on social perception distinguishes between warmth and empathy; empathy is subsumed under the umbrella of warmth as a feature that may indicate it, but other qualities that cannot be directly equated to empathy also comprise warmth (e.g., friendliness, honesty, kindness, and good-naturedness) (15). Simply being friendly or honest does not necessarily communicate empathy but could bolster perceived warmth. Thus, since it encompasses a wider variety of relevant provider characteristics and behaviors, we adopt the more general term *warmth* rather than the more specific term *empathy* in our discussion of provider qualities.

3 Effect sizes for the impact of the experimental alterations of competence and warmth on patient perceptions of providers indicated that the changes in provider behavior designed to evoke competence had a medium size effect on patient perceptions of provider competence, Cohen’s *d* = 0.47, and the changes in provider behavior designed to evoke warmth had a large effect on patient perceptions of provider warmth, Cohen’s *d* = 1.75.

## Supplementary Material

The Supplementary Material for this artcile can be found online at: https://www.frontiersin.org/articles/10.3389/fpsyt.2019.00475/full#supplementary-material
