# Supplementary material for: When Your Doctor “Gets It” and “Gets You”: The Critical Role of Competence and Warmth in the Patient–Provider Interaction
Source: Front Psychiatry. 2019 Jul 4;10:475. doi: 10.3389/fpsyt.2019.00475 (PMC6619399; doi:10.3389/fpsyt.2019.00475)
Supplement: Supplementary file 1 [file Table_1.docx]

Supplementary Material

# Supplementary Table 1

**Supplementary Table 1**. Warmth and competence in items used in patient satisfaction scales, continued from Table 4.

| Consultation Satisfaction Questionnaire (CSQ): Sample of 8 relevant items, out of 18 items | |
| --- | --- |
| **Items Associated with Competence** | **Items Associated with Warmth** |
| This doctor was very careful to check everything when examining me | This doctor was interested in me as a person, and not just my illness |
| This doctor examined me very thoroughly | This doctor knows all about me |
| This doctor told me everything about my treatment | I felt this doctor really knew what I was thinking |
|  | I felt able to tell this doctor about very personal things |
| **Items Bridging Warmth and Competence** | |
| I understand my illness much better after seeing this doctor | |
| I will follow this doctor’s advice because I think he/she is absolutely right | |
|  |  |
| Picker Patient Experience Questionnaire (PPE-15): Sample of 12 relevant items, out of 15 items | |
| **Items Associated with Competence** | **Items Associated with Warmth** |
| Sometimes in a hospital, one doctor or nurse will say one thing and another will say something quite different. Did this happen to you? | Overall, did you feel like you were treated with respect and dignity while you were in hospital? |
| Did a member of staff tell you about medication side effects to watch for when you went home? | Did the doctors talk in front of you as if you weren’t there? |
| Did someone tell you about danger signals regarding your illness or treatment to watch for after you went home? | If your family or someone else close to you wanted to talk to a doctor, did they have enough opportunity to do so? |
| **Items Bridging Warmth and Competence** | |
| When you had important questions to ask a [doctor/nurse], did you get answers that you could understand? | |
| If you had any anxieties or fears about your condition or treatment, did a [doctor/nurse] discuss them with you? | |
| Did you want to be more involved in decisions made about your care and treatment? | |
| Did a member of staff explain the purpose of the medicines you were to take at home in a way you could understand? | |
|  |  |
| Medical Interview Satisfaction Scale (MISS): Sample of 16 relevant items, out of 26 items | |
| **Items Associated with Competence** | **Items Associated with Warmth** |
| *Cognitive (9 items)* | *Affective (9 items)* |
| This doctor is very good at explaining the reasons for medical tests. | I really felt understood by my doctor. |
| The doctor told me what the medicines he prescribed for me would do for me. | I felt this doctor accepted me as a person. |
| After talking with the doctor, I know just how serious my illness is. | This doctor was not friendly to me. (R) |
| *Behavioral (8 items)* | I felt free to talk to my doctor about private thoughts. |
| The doctor gave me a thorough check-up. | I felt this doctor really knew how upset I was about my pain. |
| The doctor looked into all of the problems I mentioned. | The doctor gave me a chance to say what was really on my mind. |
| The doctor seemed to know what he was doing during the examination. |  |
| **Items Bridging Warmth and Competence** | |
| *Cognitive (9 items)* | |
| The doctor told me the name of my illness in words I could understand. | |
| The doctor told me all I wanted to know about my illness. | |
| The doctor told me how being sick will affect my ability to do work. | |
| I feel I understand pretty well the doctor’s plan for helping me. | |
|  |  |
| Short Assessment of Patient Satisfaction Scale (SAPS): 5 out of 7 items | |
| **Items Associated with Competence** | **Items Associated with Warmth** |
| How satisfied are you with the effect of your [treatment/care]? | How much of the time did you feel respected by the [doctor/other health professional]? |
| How satisfied are you with the explanations the [doctor/other health professional] has given you about the results of your [treatment/care]? |  |
| The [doctor/other health professional] was very careful to check everything when examining you. |  |
| **Items Bridging Warmth and Competence** | |
| How satisfied were you with the choices you had in decisions affecting your health care? | |

Note. (R) indicates that the item describes a provider who is lower on warmth or lower on competence. Otherwise, the item is representative of higher warmth or higher competence. Some other items in these scales not captured in this table assessed general satisfaction and/or confidence in providers, which may be shaped by perceptions of both warmth and competence.
